# General Manner of Conducting Dental Societies

**Published:** 1891-02

**Authors:** J. W. Lyder

**Affiliations:** Akron, O.


### General Manner of Conducting Dental Societies.

DR. J. W. LYDER, AKRON, O.

Read before the Akron Dental Society.

The benefits that result from association, as great as they are in commerce, politics, agricultural interests, are still greater to us as professional men. So necessary are associations to the advancement of science that the latter has rarely flourished without the aid of the former. In entering into an association new resolutions should be made, and special pledges offered to apply to each individual member. Their nature should be such as participating in all discussions, accepting any share of duty assigned us, wherein science and art are the crowning achievements in our profession.

The field which is opened up in dental association for gaining practical knowledge pertaining to our profession is unequaled in extent by any other. Our Maker has not yet created the man endowed with sufficient skill, inginuity, and brain to encompass all that is required to make a competent dentist in a short space of time. It is, therefore, of greater importance that each one avail himself of the benefits of associated labor and knowledge. The practical workings of any association should be participated in by every member. As a dormant or lifeless seed does not bring forth fruit, neither will the member who fails to engage in the discussions, or participate in the work, become a good thinker or worker. Dental associations should be so constituted as not only to educate its members, but to produce harmony and a brotherly feeling among them.

The matter of forming a schedule of prices is not a matter to be incorporated in the rules of an association. An understanding of a minimum price will be adhered to by honorable practitioners, and a good operation is worth its price, while a poor one is only worth what is charged for it, and no honorable dentist will reduce his price so as to deprive him of the best material for his operations. He should never undercharge a professional brother for the sake of drawing away his patients. This is an actual meanness which even the want of bread would not justify. It is suicidal for a man to perform cheap operations, for by the common sense of mankind he is held in cheap repute and always rated at his own price, and his public career is a short one.

The active workings of a society should be that all subjects should be assigned in advance, allowing ample time for study previous to the regular meeting. It should be a duty as well as pleasure for every member to present some new appliance or new thought at its meetings.

The adoption of a question-box has proved of great interest. The use of a blackboard is almost indispensable, and should be freely used in illustrations. The executive is the motive power, and on it depends success of development. If the above named thoughts and duties are participated in by the members of this society with zeal, we shall be better dentists, and more honorable to our profession at the close of the current year.

---

For the Hair:

R. Quiniæ sulphatis..............................x grains.

Spt. myrciæ....................................jii ounces.

Glycerinæ......................................j ounce.

Sodii chlori...................................ji drachms.

Aquæ, q. s. ad.................................jiiv ounces.

M. S. Local use. There are hundreds of so-called hair tonics containing more or less of these ingredients, but the one here given is the most satisfactory of its kind.—*Cour. of Med.*

